# The impact of patient sex on intensive care unit admission: a blinded randomized survey

**DOI:** 10.1038/s41598-019-50836-3

**Published:** 2019-10-02

**Authors:** Erik Zettersten, Gabriella Jäderling, Emma Larsson, Max Bell

**Affiliations:** 1 Perioperative Medicine and Intensive Care, Karolinska University Hospital, Solna, Stockholm Sweden; 2 Section of Anaesthesiology and Intensive Care Medicine, Department of Physiology and Pharmacology, Karolinska Institute, Stockholm, Sweden

**Keywords:** Medical ethics, Epidemiology

## Abstract

The gender distribution in intensive care units is consistently found to be around 60% men and 40% women. This might be medically sound. Our main purpose with this study was to investigate if physicians admit men and women to the intensive care unit equally. We sought to answer this question using a blinded randomized survey study. We used an online survey tool, with a hyperlink on European society of intensive care medicine webpage. Responders were randomized to either a critical care case *Jane* or a critical care case *John*, otherwise identical. The responders were asked if they would admit *Jane/John* to an intensive care unit, yes or no. Possible differences in admittance rate on the basis of the gender of the patient were analysed. In addition, we analysed if the gender of the responder affected admittance rate, regardless of the gender of the patient. 70.1% of the responders randomized to the John case opted to admit, vs. 68.3% of the responders randomized to the Jane case, p = 0.341. Regardless the gender of the patient, 70.1% of male responders opted to admit the patient, vs. 69.7% of female responders, p = 0.886. In this blinded randomized multicentre survey study, we could not demonstrate any difference in willingness to admit a patient to ICU, solely based on the gender of the patient. Patient gender as a factor for ICU admittance. A blinded randomized survey.

## Introduction

In many hospitals resources are sparse. The intensive care unit (ICU) is in a sense at the centre of this problem. Scarcity of ICU beds leads to difficult decisions regarding who to discharge and who to admit^1^. Guiding principles for the physician state that health care should be provided to all citizens equally. The person with the greatest need of care should be prioritized. It is reasonable to believe that few physicians would argue against these principles. With that in mind, it is somewhat odd that the sex distribution in ICU populations consistently display roughly 60% men and 40% women^2–4^. It might be medically correct, i.e. men need more intensive care, but could there be other explanatory factors for this disparity?

Gender refers to the socially constructed characteristics of men and women, while sex implies biological differences^5^. Gender bias, i.e. unjustified differences in the treatment of men and women depending on their sex, has been reported numerous times in different medical fields^6–11^, and may also have an impact on referral rate to ICU. Our main objective was to investigate if physicians admit men and women to the intensive care unit equally. We set out to do this by creating a survey with two identical cases, bar the sex of the patient. Responders to the survey were randomized to one of the two cases, but blinded to that. They were then asked if they would admit the patient to their ICU, a simple yes or no question. Any difference in referral rate would be attributable to the only factor distinguishing the cases, namely the sex of the patient.

## Methods

This was a randomized blinded survey study, with responders from 75 countries. It was endorsed by the European society of intensive care medicine (ESICM). The Regional Research Ethics Committee in Stockholm approved the study and all research was performed in accordance with national guidelines and regulations. Answering the survey was voluntary and informed consent was considered obtained when responders answered the survey question.

A pilot study with the same principal design was undertaken by our group in 2015^12^. Two surveys containing eight cases were sent out to physicians working in Sweden. The responders were randomized to one of the two surveys. The only difference between the two surveys was the gender of the patient in each case. In the present study we used only one.

Of the eight cases, choosing the case that in the pilot study was most contentious. The case is presented in an additional file (additional file 1).

We used an online survey tool (SurveyMonkey, SurveyMonkey INC, San Mateo, USA) with a hyperlink on the ESICM webpage. The survey was promoted with the help of ESICMs newsletter and social media channels. Unbeknown to the responders, they were randomized, using A/B Tests (Random Assignment) 50/50 randomization feature from SurveyMonkey, to either a critical care case *Jane* or a critical care case *John*, otherwise identical. At the end of the case the responders were asked if they would admit *Jane/John* to an intensive care unit, yes or no. Possible differences in admittance rate on the basis of the gender of the patient were analysed. In addition, we analysed if the gender of the responder affected admittance rate, regardless of the gender of the patient.

Data analysis was performed using Stata v.14 (Stata®, StataCorp LLC, Texas, USA). Differences in proportions were compared by Chi-square test. All tests were two-sided. A p-value of <0.05 was considered as statistically significant. For the purpose of investigating if the responder’s country of residence had any impact on admittance rate, we performed a Generalized Estimation Equation (GEE) for Logistic regression, clustering on country. The dataset analysed during the current study is available from the corresponding author on reasonable request.

## Results

The survey was online between 24^th^ November 2017 until 29^th^ January 2018 and engaged 1,004 case responders from 75 countries spread across all continents. The characteristics of the responders are presented in Table 1 and it shows that the randomization worked. There was no significant difference between *John* and *Jane* getting admitted to the ICU: John 70.1% vs. Jane 68.3% p = 0.341. The gender of the respondent did not have an impact on willingness to admit the patient to ICU, regardless of the gender of the patient: male responder 70.1% vs. female responder 69.7% P = 0.886 (Table 2). Furthermore, no difference in admittance rate between male and female patients was detected after having performed a GEE, clustering on country (OR 0.9, 95% CI: 0.7–1.2; p = 0.434).

**Table 1:** Demographics of the respondents.

Responders	Jane case	John case
Gender (n, %)		
Female	158 (32.9)	162 (32.2)
Male	323 (67.1)	341 (67.8)
Age (n, %)		
21–30	36 (7.5)	45 (9.0)
31–40>	198 (41.2)	203 (40.7)
41	247 (51.4)	251 (50.3)
Type of hospital (n, %)		
Urban	86 (17.9)	100 (20.0)
Regional	115 (23.8)	114 (22.8)
University Affiliated	282 (58.4)	287 (57.3)
Specialty (n, %)		
Anaesthesiology	276 (57.0)	298 (59.2)
Critical/Intensive Care Medicine	93 (18.4)	99 (20.4)
Cardiology	9 (1.9)	5 (1.0)
Internal medicine	55 (11.4)	59 (11.7)
Surgery	6 (1.2)	4 (0.8)
Other	45 (0.9)	38 (0.8)

**Table 2:** Female vs. male responder, regardless the gender of the patient.

Gender of Respondent	Admitting patient (n, %)	
	No	Yes
Female	97 (30.3)	223 (69.7)
Male	198 (29.7)	465 (70.1)
		p* = 0.886

*Chi-square test.

## Discussion

In this blinded randomized multicentre survey study, we could not demonstrate any difference in willingness to admit a patient to ICU, solely based on the gender of the patient. In addition, there was no significant difference between male and female physician responders in admission rate, disregarding the gender of the patient.

This study format has been applied previously. Hamberg *et al*.^7^ studied how gender of the patient would impact diagnosis and treatment in a final examination that Swedish physicians in training undertake before becoming fully licensed. Both diagnosis and treatment differed, despite the cases being identical. Their findings suggested that physicians’ stereotyped expectations are involved in creating gender differences in medicine. There are numerous examples of when reality proves them right. Arber *et al*.^8^ demonstrated that primary care doctors in the United States and United Kingdom did not ask women and elderly about risk factors for chronic heart disease as often as they asked men. An American study reported that women hospitalized for coronary heart disease underwent fewer diagnostic and therapeutic procedures than men. The authors conclude that it might represent appropriate level of care for both genders, but that it could reflect an underuse for women and overuse for men^13^. In a more recent study, Johnston *et al*.^6^ found that for patients in Canada and Sweden diagnosed with ST-elevation myocardial infarction, women were less likely than men to receive acute reperfusion therapy. Nguyen *et al*.^14^ demonstrated that women with acute myocardial infarction were to a less extent referred to angiography but received the same pharmacological treatment and had the same use of coronary revascularization as men. In other fields results are at variance. When investigating stroke care and outcome, one group in Canada found only small differences in use of statins and neuroimaging^15^. A similar European study however showed that diagnostic methods were significantly less frequently performed in female than male patients^16^.

In critical care, the results of prior research are also conflicting. In a study of 261,255 consecutive patients admitted to adult ICUs in the United States between 2004 to 2008 it was demonstrated that men received more of mechanical ventilation, emergent surgery, thrombolytic therapy and coronary artery bypass grafting. Men also had a higher readmission rate and longer length of ICU stay. However, women under 50 years of age had a lower ICU mortality than men; above 50 years of age the difference vanished^17^. In a report including 25,998 patients from 31 intensive care units in Austria no difference in mortality was noted^18^. After adjustment for age and severity of illness, men had a significantly higher probability of receiving mechanical ventilation, vasoactive medication, renal replacement therapy and a pulmonary artery catheter. Again, this suggests that men are either overusing resources, or women are underusing them. In Sweden, Samuelsson *et al*.^3^ came to similar conclusions. With 127,254 consecutive ICU admissions no survival advantage for premenopausal women was noted, but after adjusting for severity of illness and age men used more intensive care resources per admission than women did.

It seems clear that in large intensive care trials, differences in the care given *within* the ICU can be detected. It is unlikely that this is a caused by explicit gender bias, i.e. that the physician consciously neglects to treat women in the same manner as men, but it could be caused by implicit bias, which by definition is unintentional, or unconscious^19,20^. Given the results from previous research it is reasonable to assume that part of the discrepancy is caused by physicians stereotyping on gender, especially when working in a highly stressful environment such as the intensive care unit^21^. Knowing this, and keeping in mind that there is in fact a gender disparity in the ICU population, we posed the question: is the threshold for women to get access to the ICU higher? Are there women in the emergency rooms or in the wards belonging in the ICU, but not given access? We approached this question using a credible case. We had no ambition of judging if the more correct answer to our survey question is to admit or not admit. Our ambition was the opposite, to select an ambiguous case, a case that would typically be admitted by 50% and refused by 50%. We were strictly interested in deducing any difference in admittance rate between a male and female patient. Such difference would only be attributable to the one thing that separated the cases, i.e. the gender of the patient.

The strength of this study is that it tests the physician’s attitude towards admitting patients in a truly blinded randomized scenario. There is presumably no other way to do this. Using only one case, difference in admittance rate between men and women would be difficult to explain with other means than that there exists an implicit gender bias.

There are obvious limitations in this randomized survey. It is a paper case, and not a real live hospital situation. Implicit bias is “unconscious” or unintentional, and could even starkly differ from explicit beliefs^20^. In a paper case the name and gender of the patient might not be enough to trigger implicit bias in the responder. Further limitations include selection bias, people taking the time to answer surveys might not be the same people that let gender affect their clinical judgement. The response rate is also not clear, which obviously makes generalizability difficult. Moreover, as respondents came from 75 nations, presumably the heterogeneity of their workplaces is large. It was an open survey, and it would be possible for non-professionals to answer it. All respondents have declared their profession. It was possible for respondents to answer multiple times, however unlikely.

## Conclusion

In this survey study with 1,004 responders from 75 different countries, there was no difference in admittance rate that could be attributed to the gender of the patient. Furthermore, no difference was noted in admittance rate between male and female responders. These results offer no explanation to the gender discrepancy that is described in previous research^18^. To further the science in this field it is important to continue to investigate whether men and women get access to and receive the same level of care within the ICU.

## Data Availability

The dataset analysed during the current study is available from the corresponding author on reasonable request.
